# An evaluation of computational methods for aggregate data meta-analyses of diagnostic test accuracy studies

**DOI:** 10.1186/s12874-024-02217-2

**Published:** 2024-05-10

**Authors:** Yixin Zhao, Bilal Khan, Zelalem F. Negeri

**Affiliations:** Department of Statistics and Actuarial Science, University of Waterloo, 200 University Ave W, Waterloo, N2L 3G1 Ontario Canada

**Keywords:** Meta-analysis, Diagnostic test accuracy, Generalized linear mixed models, Computational methods, Adaptive Gauss-Hermite, Laplace approximation, IRLS

## Abstract

**Background:**

A Generalized Linear Mixed Model (GLMM) is recommended to meta-analyze diagnostic test accuracy studies (DTAs) based on aggregate or individual participant data. Since a GLMM does not have a closed-form likelihood function or parameter solutions, computational methods are conventionally used to approximate the likelihoods and obtain parameter estimates. The most commonly used computational methods are the Iteratively Reweighted Least Squares (IRLS), the Laplace approximation (LA), and the Adaptive Gauss-Hermite quadrature (AGHQ). Despite being widely used, it has not been clear how these computational methods compare and perform in the context of an aggregate data meta-analysis (ADMA) of DTAs.

**Methods:**

We compared and evaluated the performance of three commonly used computational methods for GLMM - the IRLS, the LA, and the AGHQ, via a comprehensive simulation study and real-life data examples, in the context of an ADMA of DTAs. By varying several parameters in our simulations, we assessed the performance of the three methods in terms of bias, root mean squared error, confidence interval (CI) width, coverage of the 95% CI, convergence rate, and computational speed.

**Results:**

For most of the scenarios, especially when the meta-analytic data were not sparse (i.e., there were no or negligible studies with perfect diagnosis), the three computational methods were comparable for the estimation of sensitivity and specificity. However, the LA had the largest bias and root mean squared error for pooled sensitivity and specificity when the meta-analytic data were sparse. Moreover, the AGHQ took a longer computational time to converge relative to the other two methods, although it had the best convergence rate.

**Conclusions:**

We recommend practitioners and researchers carefully choose an appropriate computational algorithm when fitting a GLMM to an ADMA of DTAs. We do not recommend the LA for sparse meta-analytic data sets. However, either the AGHQ or the IRLS can be used regardless of the characteristics of the meta-analytic data.

**Supplementary Information:**

The online version contains supplementary material available at 10.1186/s12874-024-02217-2.

## Background

Meta-analysis is a statistical technique used in research to combine and analyze the results of multiple independent studies on a particular topic or research question [1]. A meta-analysis of diagnostic test accuracy (DTA) is a specific type of meta-analysis that focuses on combining and analyzing data from multiple studies assessing the performance of diagnostic tests, allowing for synthesizing diagnostic test characteristics, such as sensitivity (Se) and specificity (Sp) across multiple independent studies [2, 3]. In an aggregate data meta-analysis (ADMA) of DTAs, one gathers information on the true positive (TP), true negative (TN), false positive (FP), and false negative (FN) results for a specific diagnostic test across various studies. From these data, the study-specific observed Se, Sp, and other relevant measures of diagnostic accuracy can be calculated. By pooling the results from multiple studies, researchers aim to derive summary estimates of these test characteristics, while considering the variability and potential biases present in the individual studies.

Researchers and practitioners usually use generalized linear mixed models (GLMM) such as the bivariate random-effects model of Chu and Cole [4] to meta-analyze DTA data and obtain the maximum likelihood estimates (MLEs) of the model parameters. However, unlike the linear mixed model version of Reitsma et al. (2005) [5], since Chu and Cole’s GLMM does not have a closed-form solution for the log-likelihood due to the complex random effects variance components, one needs to use numerical methods to approximate the log-likelihood function and obtain MLEs of the model parameters. Commonly used computational methods in the context of an ADMA of DTAs include the Adaptive Gaussian Hermite quadrature (AGHQ) [6], the Laplace approximation (LA) [6], and the iteratively re-weighted least squares (IRLS) [7, 8].

There have been some attempts at comparing and evaluating some of these numerical methods in different contexts. Ju et al. (2020) [9] compared the AGHQ, LA and the penalized quasi-likelihood (PQL) for meta-analyzing sparse binary data, and found that the AGHQ and PQL did not show improved performance compared to the LA. However, Ju et al. did not take the IRLS into account, and compared the numerical methods only in terms of the pooled odds ratio but not concerning the between-study variance and covariance. Additionally, their study was focused on a meta-analysis of sparse binary intervention studies outcomes, not on DTA data. Thomas, Platt & Benedetti [10] studied the performances of the PQL and AGHQ algorithm for meta-analysis of binary outcomes in the context of an individual participant data meta-analysis (IPDMA) of intervention studies. They found that there were no appreciable differences between the two computational methods. However, Thomas et al. did not consider the LA and meta-analysis of DTAs.

However, to the best of our knowledge, there was no evidence in the literature that describes the performance of these widely used computational algorithms for GLMM in the context of either IPDMA or ADMA of DTAs, partly because DTA meta-analysis is a relatively newer area of research compared to the widely studied meta-analysis of intervention studies. Additionally, since diagnosis precedes intervention, it is crucial to establish the accuracy of diagnostic tests using sound statistical methods or algorithms to minimize misdiagnosis of patients due to flawed evidence. Moreover, since meta-analytic methods for intervention or treatment studies cannot be used to meta-analyze DTA data because of differences in data characteristics and model assumptions [11], establishing evidence on the performance of computational methods for ADMA of DTA studies is needed. Therefore, this paper aims to fill this important research gap by comparing and evaluating the AGHQ, IRLS, and LA performances for GLMM to meta-analyze DTAs using aggregate data. We will compare the numerical methods using an extensive simulation study in terms of absolute bias, root mean squared error (RMSE), coverage probability, 95% confidence interval (CI) width, convergence rate, and computational speed. We will also illustrate the methods using real-life meta-analytic data.

The rest of this article is organized as follows. Motivating examples section presents motivating examples using two real-life data, Methods section introduces the statistical methods, including the GLMM model, the numerical algorithms and a simulation study. In Simulation study results section, we discuss our simulation study results, and in Illustrative examples section, we illustrate the computational methods using the motivating examples data. We conclude the manuscript with a discussion and concluding remarks in Discussion and Conclusions sections.

## Motivating examples

This Section describes two real-life data sets (see Appendix Tables A1 and A2) to motivate the statistical methods we present in Methods section.

**Fig. 1:**
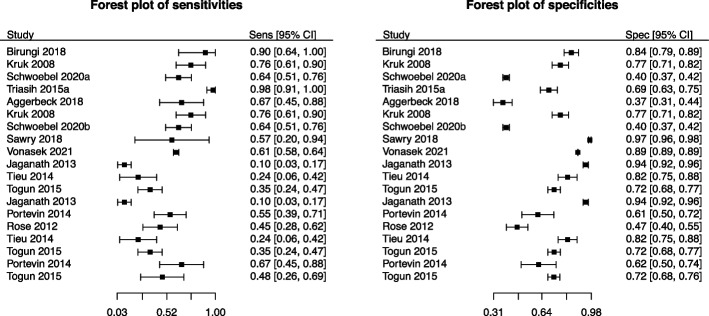
Forest plots of sensitivity (left) and specificity (right) of the meta-analysis from Vonasek et al. (2021) [12]. The a and b in Schwoebel 2020 denote the two distinct screening tests, “One or more of cough, fever, or poor weight gain in tuberculosis contacts” and “One or more of cough, fever, or decreased playfulness in children aged under five years, inpatient or outpatient,” respectively, utilized in the study

First, consider an article by Vonasek et al. (2021) [12], which studied the accuracy of screening tests (e.g., visually identifying early signs and symptoms) for active pulmonary tuberculosis in children. Figure 1 depicts the forest plots of the sensitivity and specificity measurements.

The meta-analysis of Vonasek et al. [12] included 19 studies with no indication of sparsity in either Se or Sp; that is, none of the included primary studies had observed Se or Sp close to 0 or 1. The average number of diseased ($n_1$) and non-diseased ($n_2$) participants were about 99 and 11,058, respectively, where the average $n_2$ was affected by four potentially outlier studies whose respective number of non-diseased participants were 1,903 [13], 1,903 [13], 1,336 [14], and 200,580 [15]. In Illustrative examples section, we will demonstrate how the three computational algorithms deal with the data since the existence of such outlying studies may potentially distort the meta-analysis results.

**Fig. 2:**
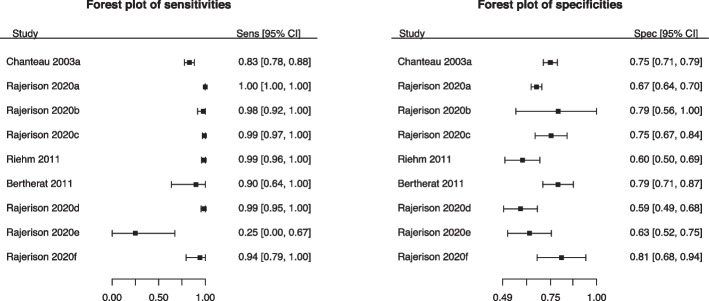
Forest plots of sensitivity (left) and specificity (right) of the meta-analysis from Jullien et al. (2020) [16]

In the second example, we present the study by Jullien et al. (2020) that studied the diagnosing characteristics of “Rapid diagnostic tests for plague” [16]. As can be seen from the forest plots presented in Fig. 2, this meta-analysis contained only nine studies and the average number of diseased and non-diseased participants were 188 and 223, respectively, with no indication of potentially outlying studies.

However, the second meta-analysis had some sparse data, particularly in the diseased group. There were 4/9 (44%) primary studies with 100% sensitivity (i.e., with $FN=0$). Thus, we will revisit this data set in Illustrative examples section to examine how the numerical methods perform in the context of sparse DTAs.

## Methods

In this Section, we describe the commonly used conventional meta-analytic model for ADMA of DTAs, the three computational methods used to estimate the parameters of this model and methods for our simulation study.

### The standard model

The bivariate binomial-normal (BBN) model is a bivariate random-effects model first developed by Chu and Cole [4]. The BBN model assumes the binomial distribution for modelling the within-study variability and the bivariate normal distribution for modelling the between-study variability in Se and Sp across studies. The BBN is generally accepted as the preferred model for ADMA of DTAs because it models the within-study variability using the exact Binomial distribution, instead of approximating it with the normal distribution, and it does not require an ad hoc continuity correction when any of the four cell frequencies in a DTA contain zero counts. If we let $\textbf{y}_i = [\text {logit}(Se_i), \text {logit}(Sp_i)]'$ denote the study-specific logit-transformed sensitivity and specificity vector, $\textbf{b}_i$ the study-specific random-effects, $\varvec{\mu }$ the pooled sensitivity and specificity vector, and $\varvec{\Sigma }$ the between-study heterogeneity parameter, the marginal likelihood function of the BBN model can be given as in equation 1. However, since this likelihood does not have closed-form expression because the integral cannot be evaluated analytically in a closed-form [4], one needs to use numerical approximation methods to estimate the likelihood.

$$\begin{aligned} TP_i|b_{1i}&\sim \text {Binomial}(n_{1i}, Se_i); y_{1i} = \mu _1 + b_{1i}; \\ TN_i|b_{2i}&\sim \text {Binomial}(n_{2i}, Sp_i); y_{2i} = \mu _2 + b_{2i}; \\ \textbf{b}_i&\sim N_2(\textbf{0}, \varvec{\Sigma }); \end{aligned}$$

1 $$\begin{aligned} L(\varvec{\mu }, \varvec{\Sigma }|\textbf{y}) = \int _{\mathbb {R}^2}\prod _{i=1}^{k}f_{\mathbf {y_i}| \textbf{b}_i}(\textbf{y}_i|\textbf{b}_i,\varvec{\mu })f_{\textbf{b}_i}(\textbf{b}_i|\mathbf {\Sigma }_i)d\textbf{b}_i, \end{aligned}$$

where $i=1,...,k$ denotes the *i*-th study in the meta-analysis.

The AGHQ [6] is a numerical method used to approximate log-likelihoods by numerical integration to obtain the MLEs of model parameters. Although estimation becomes more precise as the number of quadrature points increases, it often gives rise to computational difficulties for high-dimension random effects and convergence problems where variances are close to zero or cluster sizes are small [6]. Most of the time, the AGHQ [6] is the default estimation method and is regarded as the most accurate. Nonetheless, the LA [6] which is the Gauss-Hermite quadrature of order one [17] and the IRLS [7, 8] that aims to find the solution to a weighted least squares iteratively, can also be used to find MLEs and usually have lower computational difficulties and faster computational speed.

### Simulation study design

#### Data simulation

To compare the three computational methods for each combination of model parameter settings, we simulated data based on each simulation scenario and fitted the BBN model using the AGHQ, LA, and IRLS algorithms. To inform our simulations, we scraped the Cochrane Database of Systematic Reviews and selected 64 reviews containing meta-analyses data. Unwrapping these reviews and performing data cleaning gave us access to 393 meta-analyses covering a wide range of medical diagnosis tests. We fitted the BBN model to each of the 393 meta-analyses to obtain the empirical distribution of the model parameters. Based on these results, we defined our true parameter settings as shown in Table 1. Following Ju et al. (2020) [9] and Jackson et al. (2018) [18], we introduced sparsity into the meta-analysis by considering large values of (*Se*, *Sp*).

**Table 1:** True parameter settings for the simulation study

Parameter	Setting 1	Setting 2	Setting 3	Setting 4
(*Se*, *Sp*)	(0.7, 0.8)	(0.8, 0.9)	(0.95, 0.99)	
$(\sigma _{1}^2, \sigma _{2}^2)$	(0.90, 0.55)	(1.51, 1.0)	(1.59, 1.83)	
$\sigma _{12}$	-0.03	-0.34	-0.70	
$(n_1, n_2)$	(50,100)	(100,200)	(200,300)	
*k*	5	15	25	50

Accordingly, we considered a total of $3^4\times 4 = 324$ total scenarios in our simulation study. For each parameter combination, we conducted our simulation study by (1) simulating 1000 DTA data based on normal random effects following the steps described by Negeri and Beyene [19], (2) fitting the BBN model to each simulated data using the three computational methods, and (3) comparing the estimated results by each numerical method with the true values in terms of absolute bias, RMSE, CI width, coverage probability, convergence rate, and computing time.

We used the R statistical language [20] version 4.2.2 and RStudio [21] version 2023.09.0+463 for all data analyses. We utilized the glmer() function from the lme4 R package [22] to apply the IRLS and LA by setting nAGQ to 0 and 1, respectively. We fitted the BBN model with the AGHQ algorithm using the mixed_model() function from the GLMMadaptive R package [23] by setting the number of quadrature points used in the approximation (nAGQ) to 5.

#### Performance evaluation criteria

In our simulation study, we defined the convergence rate of the BBN model as the number of converged fits over the total number of fits in an iteration. We counted fits with non-positive semi-definite covariance matrices and fits that did not meet optimality conditions as non-converging. While assessing the convergence rate, we found that the “converged” message provided in the model summary from the glmer() function is sometimes non-trustable. For example, we saw a warning message such as “boundary (singular) fit: see help(’isSingular’)” when fitting the BBN model, which indicates a fit that did not converge, but the “converged” option wrongly provided convergence. Thus, we treated those singular fits as non-convergence to calculate the convergence rate. We measured the computing speed for each numerical method using R’s built-in function system.time(). The remaining metrics, such as the absolute bias, RMSE, coverage probability, and CI width were calculated following Burton et al. (2006) [24] and Morris et al. (2019) [25].

## Simulation study results

In this Section, we use the different metrics described in Methods section to evaluate the performance of the three computational methods and summarize our simulation study findings by metrics. Note that the solid line is IRLS, the dashed line is LA, the dotted line is AGHQ, and that the lines might overlap for some scenarios when there is no difference in results between the three computational methods.

### Absolute bias

**Fig. 3:**
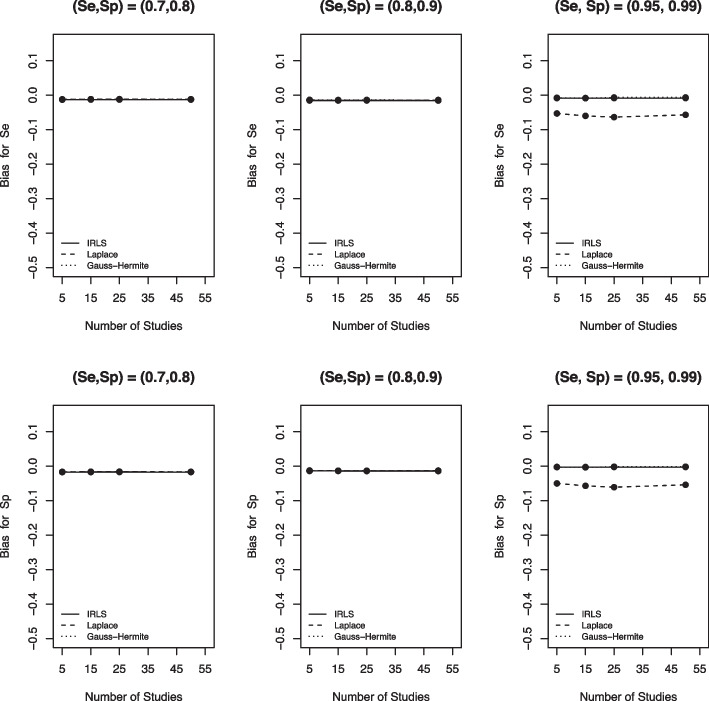
Bias for sensitivity (Se) and specificity (Sp) based on the IRLS (solid line), Laplace approximation (dashed line) and Gauss-Hermite quadrature (dotted line) when $\sigma _1^2=1.59$, $\sigma _2^2=1.83$, $\sigma _{12}=-0.34$, $n_1=300$, and $n_2=500$

Figure 3 depicts the bias of the three computational methods for sensitivity and specificity. We found that when the true Se and Sp were far from perfect, there was barely any difference among these three numerical methods as the three lines overlap for the first two columns. However, for all variance-covariance settings, the LA had the largest absolute bias compared to the AGHQ and the IRLS (Fig. 3, third pane). Moreover, when data is sparse (i.e. large Se and Sp closer to 100%), the IRLS and AGHQ were comparable, although IRLS had a slightly larger absolute bias. We observed consistent results for the other scenarios considered in our simulations (see the Appendix figures).

**Fig. 4:**
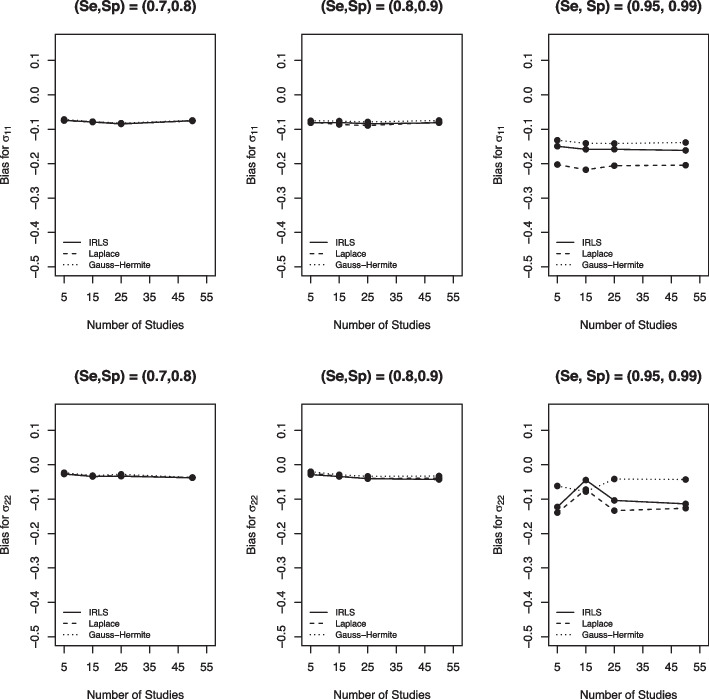
Bias for between-study variances based on the IRLS (solid line), Laplace approximation (dashed line) and Gauss-Hermite quadrature (dotted line) when $\sigma _1^2=1.59$, $\sigma _2^2=1.83$, $\sigma _{12}=-0.34$, $n_1=300$, and $n_2=500$

Similarly, the three computational methods had comparable performance when it comes to the bias of the between-study variances $\sigma _{1}^2$ and $\sigma _{2}^2$ for relatively small Se and Sp (Fig. 4, first two panes). However, for sparse DTA data (large Se and Sp), the LA still had the largest absolute bias, and the AGHQ had the smallest bias for between-study variances. Similar results were found for the other scenarios examined in our simulations (see the Appendix figures).

### Root mean squared error (RMSE)

**Fig. 5:**
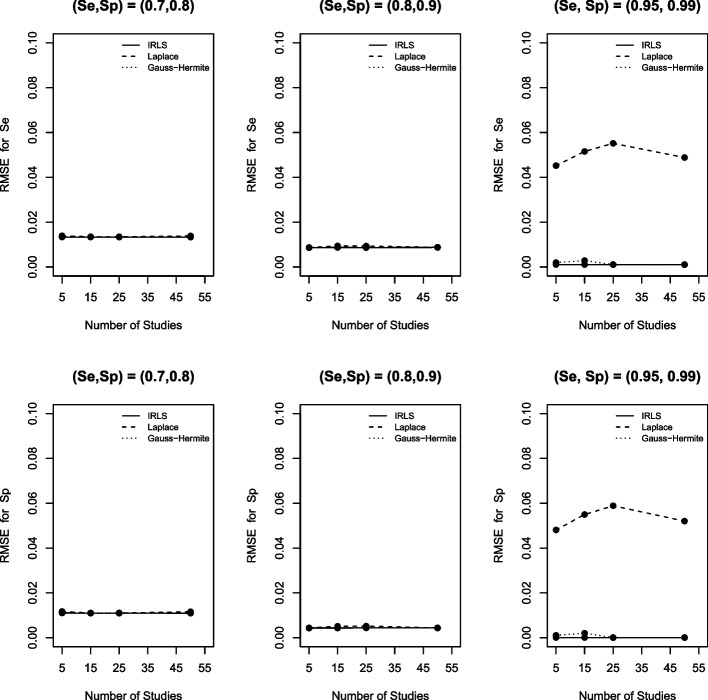
RMSE for sensitivity (Se) and specificity (Sp) based on the IRLS (solid line), Laplace approximation (dashed line) and Gauss-Hermite quadrature (dotted line) when $\sigma _1^2=1.59$, $\sigma _2^2=1.83$, $\sigma _{12}=-0.34$, $n_1=300$, and $n_2=500$

Concerning RMSE (Fig. 5), we observed a similar trend to bias. That is, the three numerical methods were comparable when the DTA data was not sparse, but the LA yielded larger RMSE for all (Se, Sp) pairs. Furthermore, the IRLS and the AGHQ were comparable, although the AGHQ had a slightly larger RMSE. Consistent results were observed for the other scenarios considered in our simulations (see the Appendix figures).

### Confidence interval (CI) width and coverage

**Fig. 6:**
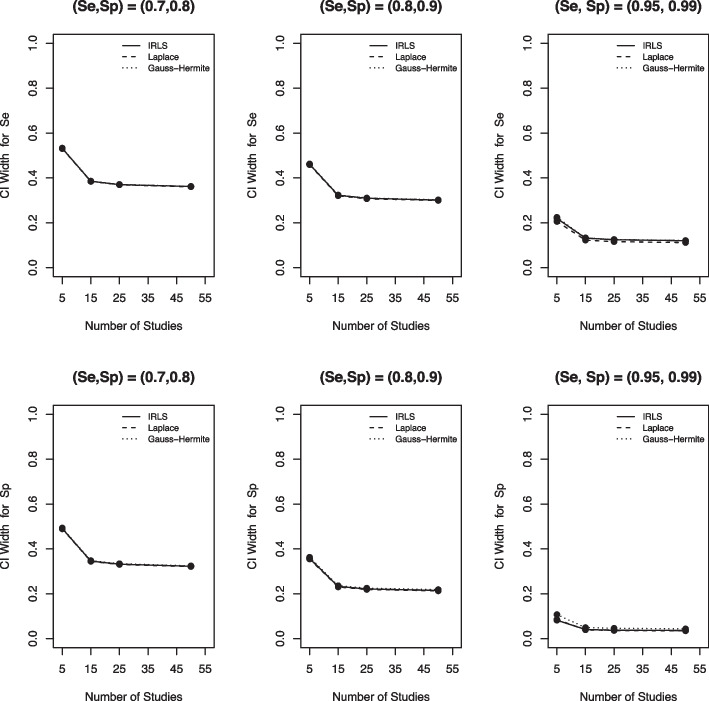
CI width for sensitivity (Se) and specificity (Sp) based on the IRLS (solid line), Laplace approximation (dashed line) and Gauss-Hermite quadrature (dotted line) when $\sigma _1^2=1.59$, $\sigma _2^2=1.83$, $\sigma _{12}=-0.34$, $n_1=300$, and $n_2=500$

For CI width (Fig. 6), the three numerical methods gave almost the same results when the true Se and Sp were small. However, there were marginal differences among the computational methods when DTA was sparse, as the IRLS had the smallest CI width for specificity and the LA yielded the smallest CI width for sensitivity. Moreover, as Se or Sp increased, the width of the CI decreased.

**Fig. 7:**
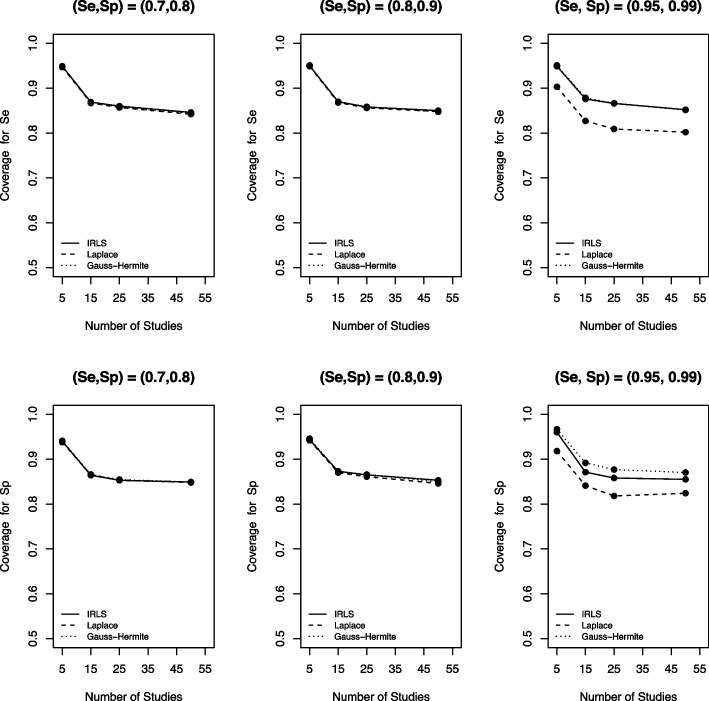
Coverage for sensitivity (Se) and specificity (Sp) based on the IRLS (solid line), Laplace approximation (dashed line) and Gauss-Hermite quadrature (dotted line) when $\sigma _1^2=1.59$, $\sigma _2^2=1.83$, $\sigma _{12}=-0.34$, $n_1=300$, and $n_2=500$

Figure 7 presents the coverage probabilities of the three computational methods. Similar to the other metrics, the AGHQ, LA, and IRLS had comparable coverage probability when data were not sparse (i.e., small Se and Sp). However, the LA had the smallest coverage probability for sparse DTA data compared to the other two methods, and the AGHQ had a slightly larger coverage than the IRLS. Moreover, as the number of studies in a meta-analysis increased, the coverage probability of the methods decreased. We found similar results for the other simulation scenarios considered in our simulations (see the Appendix figures).

### Convergence rate and computing time

Table 2 depicts the average convergence rate, average computing time, and the interquartile range (IQR) for computing time across all simulation scenarios for the three computational methods. Accordingly, on average, the AGHQ had the highest convergence rate but the longest computing time compared to the two methods. We also observed that longer computing times were associated with higher convergence rates. Moreover, the AGHQ also had the largest IQR of the three numerical methods.

**Table 2:** Average convergence rate, average computing time, and IQR computing time by computational method

Performance Metric	IRLS	LA	AGHQ
Convergence Rate	$69.75\%$	$73.33\%$	$98.14\%$
Average Computing Time (seconds)	0.0782	0.1670	0.4860
IQR Computing Time (seconds)	0.0012	0.0130	0.3900

## Illustrative examples

This Section summarizes the results of fitting the BBN model to the two motivating examples presented in Motivating examples section using the three computational algorithms.

Table 3 summarizes the results of applying the numerical algorithms to the Vonasek et al. (2021) [12] data. All three numerical algorithms converged to the MLEs. The AGHQ estimated both the pooled Se and pooled Sp very differently than the other two methods. The LA and IRLS approaches resulted in similar pooled Se and pooled Sp estimates, with their pooled Sp closer to the observed specificities of the outlying studies identified in Motivating examples section than the non-outlying studies, indicating that the LA and IRLS estimates may be influenced by outlying studies [2, 3]. These results suggest that the AGHQ yielded estimates that were less affected by the outlying studies in specificity. However, all three methods yielded comparable between-study variance-covariance estimates.

**Table 3:** Application of the three computational methods to the Vonasek et al. (2021) [12] meta-analysis

Method	Se (95% CI)	Sp (95% CI)	$\varvec{\sigma }_{\varvec{1}}^{\varvec{2}}$	$\varvec{\sigma }_{\varvec{12}}$	$\varvec{\sigma }_{\varvec{2}}^{\varvec{2}}$	Conv
AGHQ	0.3880 (0.0729, 0.8364)	0.8996 (0.1357, 0.9980)	1.1350	-0.6005	1.2299	Yes
LA	0.5135 (0.3668, 0.6579)	0.7551 (0.6446, 0.8398)	1.1310	-0.5949	1.2075	Yes
IRLS	0.5119 (0.3668, 0.6550)	0.7540 (0.6433, 0.8390)	1.1301	-0.5952	1.2074	Yes

We present the results of fitting the BBN model to the meta-analysis of Jullien et al. (2020) [16] in Table 4. The AGHQ algorithm failed to converge with its Hessian matrix being non-positive-definite. Despite that, all three methods produced comparable pooled Se and Sp estimates, $\sigma _{12}$ and $\sigma _2^2$. However, the LA produced a very large between-study variance of logit-transformed sensitivity $(\sigma _1^2)$, which could be attributed to the apparent data sparsity among the diseased participants, consistent with our simulation study results.

**Table 4:** Application of the three computational methods to the Jullien et al. (2020) [16] meta-analysis

Method	Se (95% CI)	Sp (95% CI)	$\varvec{\sigma }_{\varvec{1}}^{\varvec{2}}$	$\varvec{\sigma }_{\varvec{12}}$	$\varvec{\sigma }_{\varvec{2}}^{\varvec{2}}$	Conv
AGHQ	0.9975 (NA, NA)	0.7035 (0.637, 0.7623)	9.7051	-0.1815	0.0548	Yes^a^
LA	1.0000 (0.6599, 1)	0.7033 (0.638, 0.7611)	27.2992	-0.3001	0.0559	Yes
IRLS	0.9955 (0.4965, 1)	0.7019 (0.638, 0.7588)	9.7716	-0.1652	0.0544	Yes

^a^Hessian is non-positive-definite, hence invalid CI limits

## Discussion

In this study, we compared three commonly used computational algorithms, the AGHQ, the LA, and the IRLS, that numerically approximate the log-likelihood function of a bivariate GLMM for ADMA of DTAs. To determine which method is more appropriate in practice, we compared the performance of these methods using extensive simulation studies and real-life data sets. Our simulation settings were informed after analyzing 393 real-life meta-analyses from the Cochrane Database of Systematic Reviews.

In almost all of our simulation scenarios, we observed no obvious difference among the three numerical methods when Se and Sp were relatively small and not close to 100%. However, when the DTA data were sparse or equivalently when Se and Sp were both large and close to 100%, there were appreciable differences among these three computational algorithms. The LA usually had the largest absolute bias and RMSE but the smallest coverage probability for Se and Sp compared to the IRLS and the AGHQ. The IRLS and AGHQ were comparable, but IRLS had the smallest convergence rate. Though the AGHQ had the largest convergence rate among the three algorithms, it had the longest computing time.

Unlike the results reported by Ju et al. (2020) [9] for meta-analysis of rare intervention studies, we found appreciable differences in bias and RMSE of the LA and the AGHQ for sparse data, albeit in the context of ADMA of DTAs. However, we were not able to make similar comparisons in terms of the between-study variances since it wasn’t reported in their study. Similarly, a comparison was impossible between our findings and those of Thomas et al. (2017) [10] since the latter study evaluated only the AGHQ, not the LA and IRLS algorithms.

Our real-life data analyses also revealed consistent results with our simulation studies. The AGHQ produced robust pooled Se and Sp estimates when applied to DTA data with a few outlying studies. The LA yielded the largest between-study variance estimates when a GLMM was fitted to sparse DTA data. Although the PQL approach has been discouraged by other researchers in the context of intervention studies meta-analysis with binary outcomes [9] and is not commonly used in the context of meta-analysis of DTA studies, following a Reviewer’s suggestion, we applied it to our motivating examples data sets (see Appendix Table C3) and observed inferior results consistent with that of Ju et al. [9]. Thus, we opted not to investigate its performance in our simulation study. Moreover, it was not unexpected to find the LA and IRLS algorithms affected by outliers since they utilize methods known to be prone to unusual observations – the normal distribution and least squares, respectively. Whereas the LA works by approximating the *integrand* of the likelihood with the normal distribution, for example, the IRLS iteratively solves a system of score equations via weighted least squares. The AGHQ approximates the entire likelihood or *integral* via a numerical approach known as quadrature method, making it the least sensitive approach to outliers.

The strengths of our manuscript include being the first study to report on the evaluation and comparison of commonly used computational methods for ADMA of DTAs and considering several real-life scenarios by informing our simulation study with 393 meta-analysis results from the Cochrane Database of Systematic Reviews. Thus, our study has contributed to the literature by filling an existing gap in the body of knowledge and by producing results applicable to practical real-world situations. Although we considered only the frequently used numerical methods in ADMA of DTAs, not including more than three such computational algorithms can be considered a limitation of our study, which can be pursued in a future study. For example, it is worth evaluating and validating the performance of these numerical methods in comparison with the Newton-Raphson-based algorithms [26], the many procedures implemented in the *metadta* Stata tool [27], or in the context of IPDMA of DTA studies with or without multiple cut-offs [28]. Moreover, the LA and IRLS algorithms appeared to be impacted by outlying studies when applied to a real-life meta-analysis. Thus, it is worth a future study investigating this issue further via a simulation study to see if this property of the two algorithms repeats for different data settings.

## Conclusions

In summary, the IRLS, AGHQ, and the LA had similar performances for non-sparse data, but the LA performed worse for sparse DTA data sets. Whereas the AGHQ had the best convergence rate but the longest computing time, the IRLS had the shortest computing time but the worst convergence rate. Therefore, we suggest practitioners and researchers use any of the three computational methods for conducting ADMA of DTAs without sparse data. However, the LA should be avoided and either the IRLS or the AGHQ should be used when sparsity is a concern.

### Supplementary Information

**Additional file 1.** Absolute bias, RMSE, CI width, and coverage probabilities of the three computational methods for additional simulation scenarios.

## Data Availability

All data generated or analyzed during this study will be included in this published article and its supplementary information files.

## Abbreviations

ADMA: Aggregate Data Meta-Analysis
AGHQ: Adaptive Gaussian-Hermite Quadrature
BBN: Bivariate Binomial-Normal
CI: Confidence Interval
DTA: Diagnostic Test Accuracy
GLMM: Generalized Linear Mixed Models
IPDMA: Individual Participant Data Meta-Analysis
IQR: Interquartile Range
IRLS: Iteratively Reweighted Least Squares
LA: Laplace Approximation
PQL: Penalized Quasi-likelihood
RMSE: Root Mean Squared Error
Se: Sensitivity
Sp: Specificity
