# Supplementary material for: An evaluation of computational methods for aggregate data meta-analyses of diagnostic test accuracy studies
Source: BMC Med Res Methodol. 2024 May 10;24:111. doi: 10.1186/s12874-024-02217-2 (PMC11084104; doi:10.1186/s12874-024-02217-2)
Supplement: Supplementary file 1 — Additional file 1. Absolute bias, RMSE, CI width, and coverage probabilities of the three computational methods for additional simulation scenarios. [file 12874_2024_2217_MOESM1_ESM.pdf]

An appendix to "An evaluation of computational methods for aggregate data meta-analyses of diagnostic test accuracy studies" by Yixin Zhao, Bilal Khan, and Zelalem F. Negeri

Appendix A Application of the PQL algorithm to the two motivating meta-analyses

Table A1: Aggregate DTA data for the Vonasek et al. (2021) meta-analysis. n_1 (TP+FN) and n_2 (TN+FP) denote the number of diseased and non-diseased participants, respectively.

Author	n_1	TP	FN	n_2	TN	FP
Birungi 2018	4	4	0	212	179	33
Kruk 2008	33	25	8	219	168	51
Schwoebel 2020	55	35	20	1903	753	1150
Triasih 2015a	21	21	0	248	171	77
Aggerbeck 2018	18	12	6	217	81	136
Kruk 2008	33	25	8	219	168	51
Schwoebel 2020	55	35	20	1903	753	1150
Sawry 2018	7	4	3	1336	1295	41
Vonasek 2021	1212	742	470	200580	178099	22481
Jaganath 2013	79	8	71	682	642	40
Tieu 2014	21	5	16	137	112	25
Togun 2015	62	22	40	418	303	115
Jaganath 2013	79	8	71	682	642	40
Portevin 2014	38	21	17	75	46	29
Rose 2012	33	15	18	178	84	94
Tieu 2014	21	5	16	137	112	25
Togun 2015	62	22	40	418	303	115
Portevin 2014	18	12	6	63	39	24
Togun 2015	21	10	11	459	332	127

Table A2: Aggregate DTA data for the Jullien et al. (2020) meta-analysis. n_1 (TP+FN) and n_2 (TN+FP) denote the number of diseased and non-diseased participants, respectively.

Author	n_1	TP	FN	n_2	TN	FP
Chanteau 2003a	182	151	31	509	381	128
Rajerison 2020a	1328	1328	0	991	667	324
Rajerison 2020b	22	22	0	11	9	2
Rajerison 2020c	57	57	0	94	71	23
Riehm 2011	47	47	0	102	61	41
Bertherat 2011	4	4	0	92	73	19
Rajerison 2020d	40	40	0	109	64	45
Rajerison 2020e	4	1	3	63	40	23
Rajerison 2020f	8	8	0	33	27	6

Appendix B Complete Results for Simulation Data

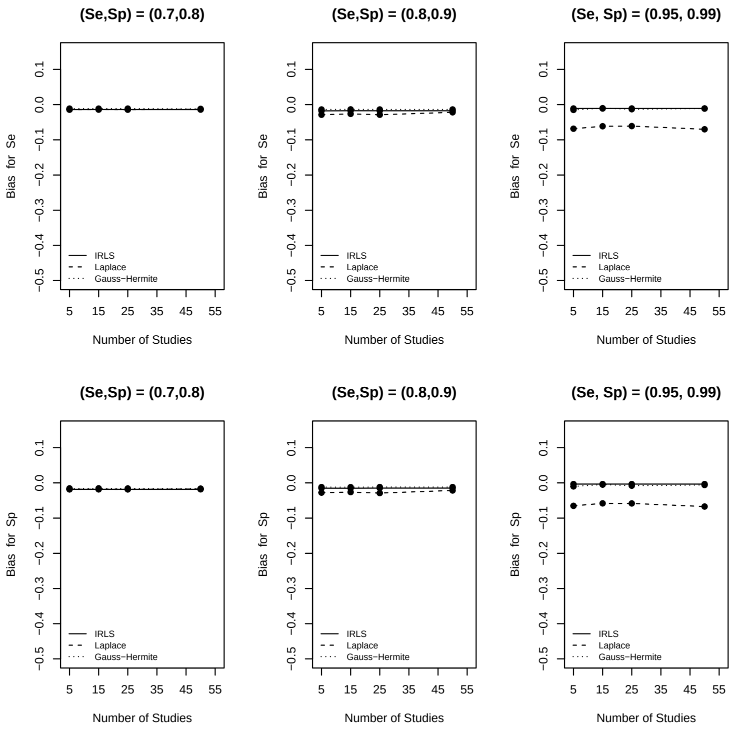

Fig. B1: Bias for sensitivity (Se) and specificity (Sp) based on the IRWLS (solid line), Laplace approximation (dashed line) and Gauss-Hermite quadrature (dotted line) when $\sigma_1^2 = 1.59$, $\sigma_2^2 = 1.83$, $\sigma_{12} = -0.34$, $n_1 = 100$, and $n_2 = 200$

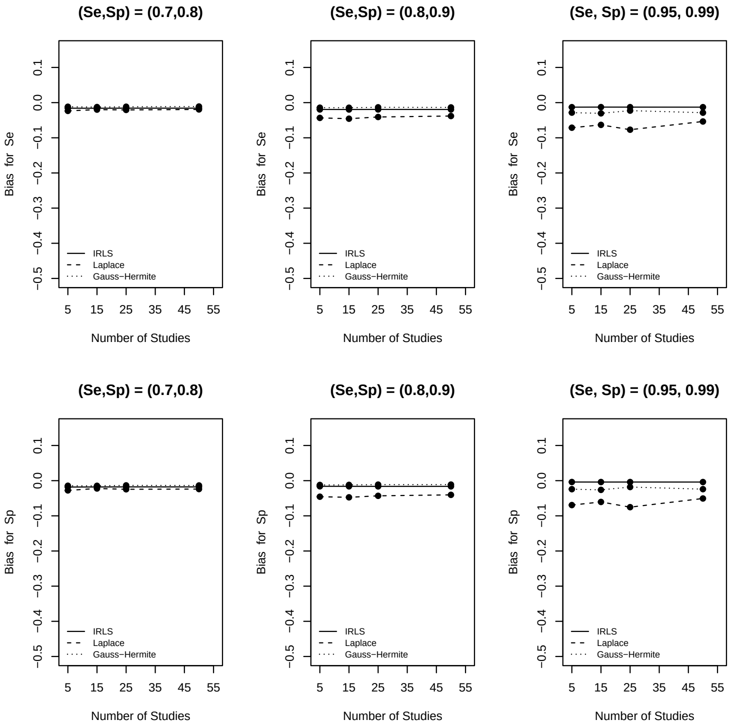

Fig. B2: Bias for sensitivity (Se) and specificity (Sp) based on the IRWLS (solid line), Laplace approximation (dashed line) and Gauss-Hermite quadrature (dotted line) when $\sigma_1^2 = 1.59$, $\sigma_2^2 = 1.83$, $\sigma_{12} = -0.34$, $n_1 = 50$, and $n_2 = 100$

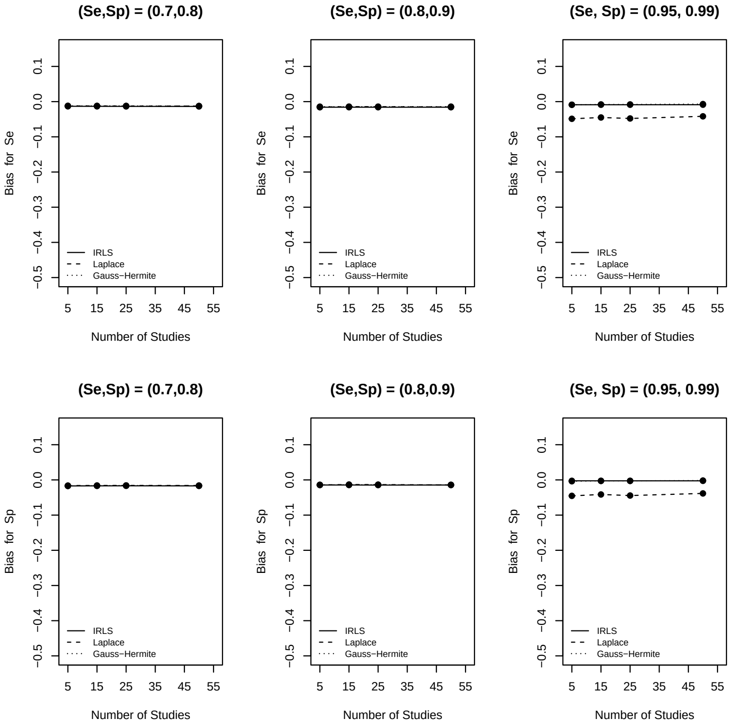

Fig. B3: Bias for sensitivity (Se) and specificity (Sp) based on the IRWLS (solid line), Laplace approximation (dashed line) and Gauss-Hermite quadrature (dotted line) when $\sigma_1^2 = 1.59$, $\sigma_2^2 = 1.83$, $\sigma_{12} = -0.7$, $n_1 = 300$, and $n_2 = 500$

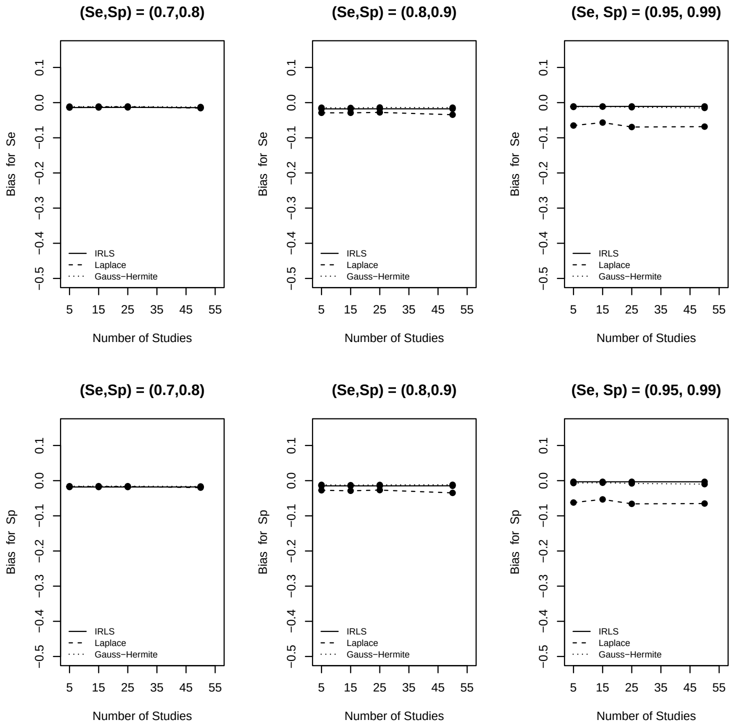

Fig. B4: Bias for sensitivity (Se) and specificity (Sp) based on the IRWLS (solid line), Laplace approximation (dashed line) and Gauss-Hermite quadrature (dotted line) when $\sigma_1^2 = 1.59$, $\sigma_2^2 = 1.83$, $\sigma_{12} = -0.7$, $n_1 = 100$, and $n_2 = 200$

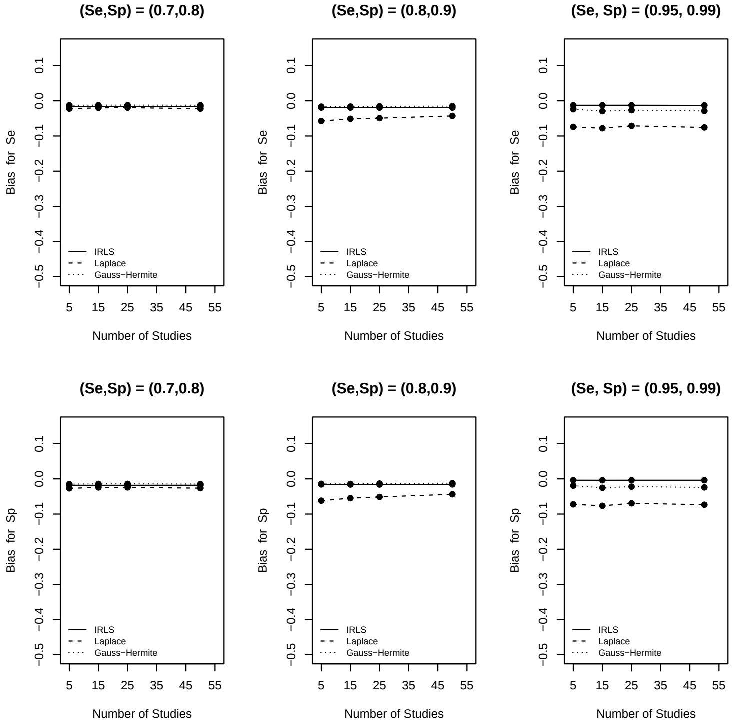

Fig. B5: Bias for sensitivity (Se) and specificity (Sp) based on the IRWLS (solid line), Laplace approximation (dashed line) and Gauss-Hermite quadrature (dotted line) when $\sigma_1^2 = 1.59$, $\sigma_2^2 = 1.83$, $\sigma_{12} = -0.7$, $n_1 = 50$, and $n_2 = 100$

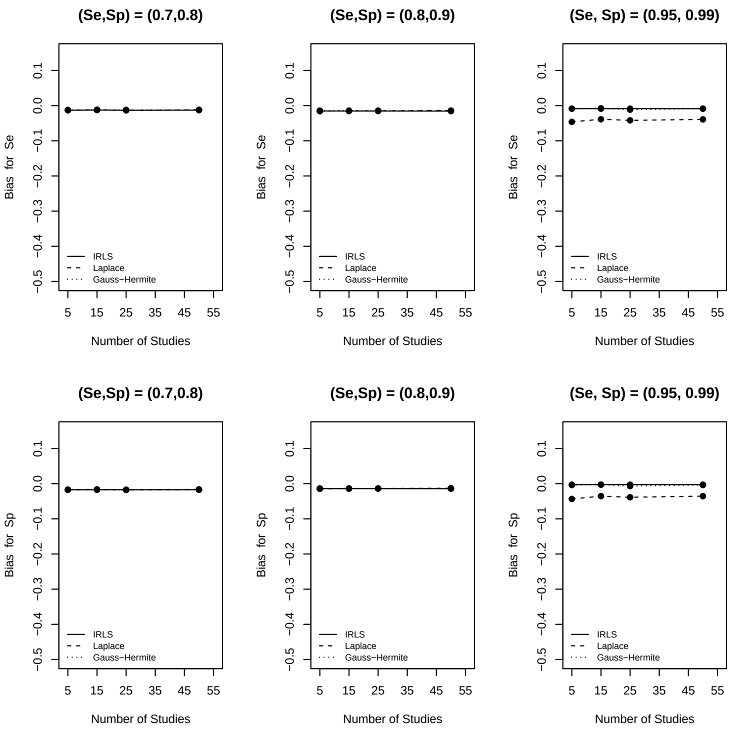

Fig. B6: Bias for sensitivity (Se) and specificity (Sp) based on the IRWLS (solid line), Laplace approximation (dashed line) and Gauss-Hermite quadrature (dotted line) when $\sigma_1^2 = 1.59$, $\sigma_2^2 = 1.83$, $\sigma_{12} = -0.03$, $n_1 = 300$, and $n_2 = 500$

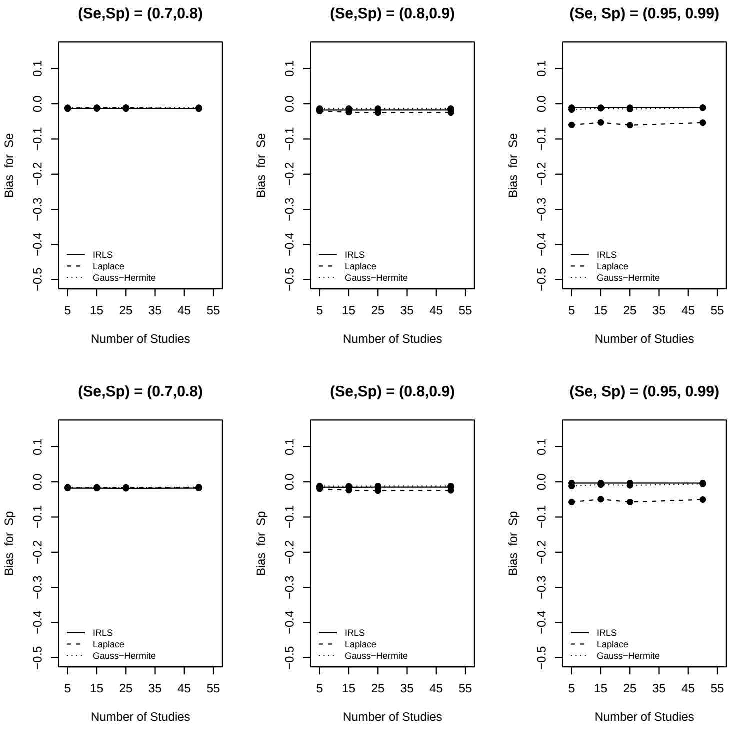

Fig. B7: Bias for sensitivity (Se) and specificity (Sp) based on the IRWLS (solid line), Laplace approximation (dashed line) and Gauss-Hermite quadrature (dotted line) when $\sigma_1^2 = 1.59$, $\sigma_2^2 = 1.83$, $\sigma_{12} = -0.03$, $n_1 = 100$, and $n_2 = 200$

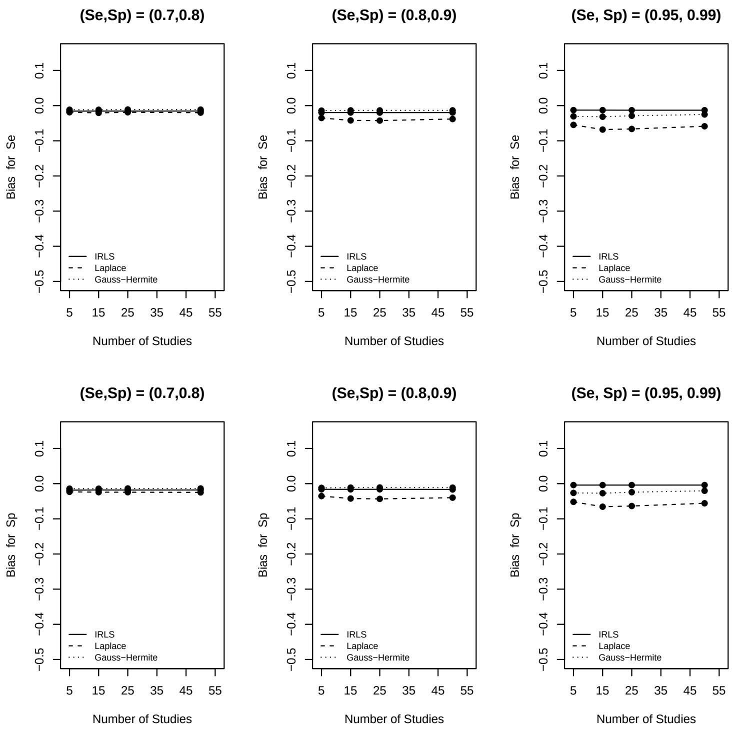

Fig. B8: Bias for sensitivity (Se) and specificity (Sp) based on the IRWLS (solid line), Laplace approximation (dashed line) and Gauss-Hermite quadrature (dotted line) when $\sigma_1^2 = 1.59$, $\sigma_2^2 = 1.83$, $\sigma_{12} = -0.03$, $n_1 = 50$, and $n_2 = 100$

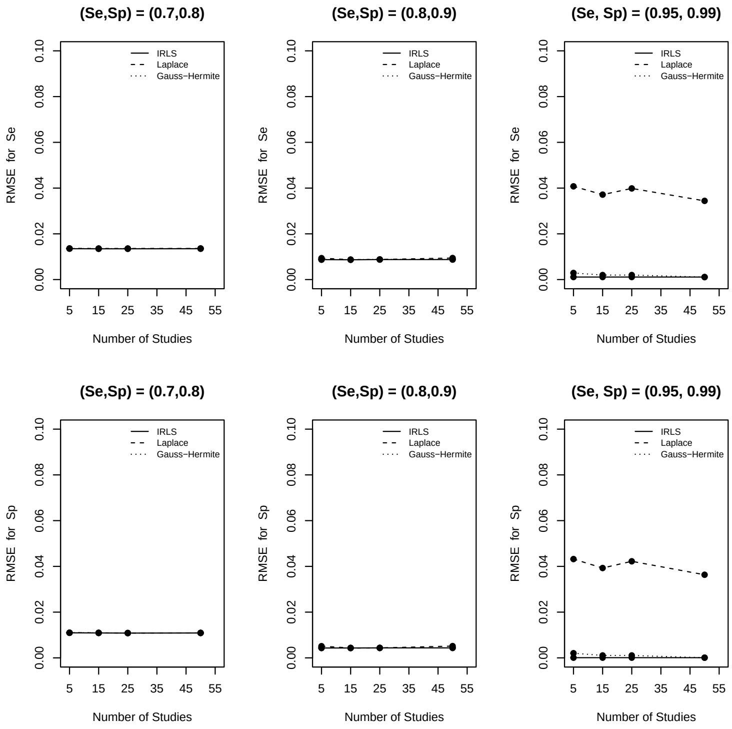

Fig. B9: RMSE for sensitivity (Se) and specificity (Sp) based on the IRWLS (solid line), Laplace approximation (dashed line) and Gauss-Hermite quadrature (dotted line) when $\sigma_1^2 = 1.59$, $\sigma_2^2 = 1.83$, $\sigma_{12} = -0.7$, $n_1 = 300$, and $n_2 = 500$

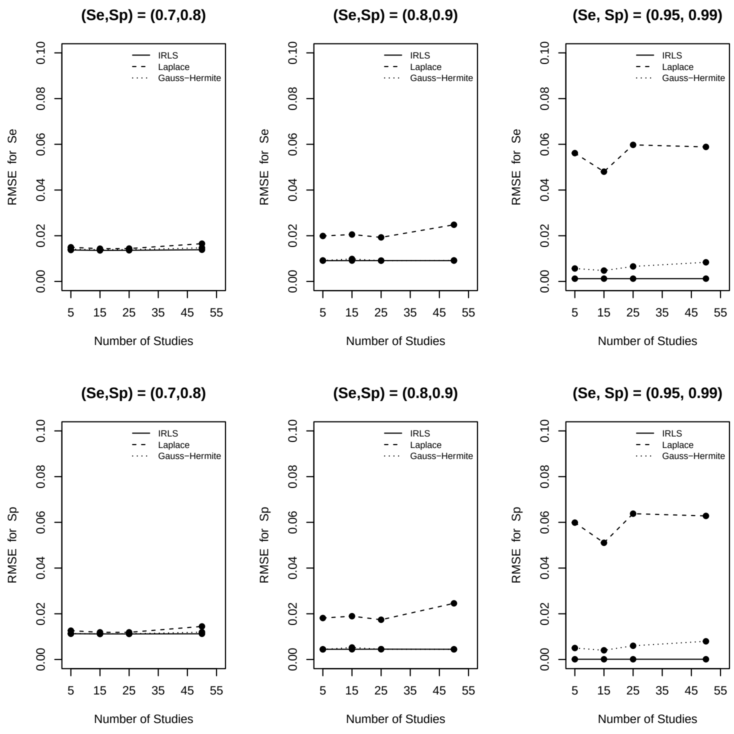

Fig. B10: RMSE for sensitivity (Se) and specificity (Sp) based on the IRWLS (solid line), Laplace approximation (dashed line) and Gauss-Hermite quadrature (dotted line) when $\sigma_1^2 = 1.59$, $\sigma_2^2 = 1.83$, $\sigma_{12} = -0.7$, $n_1 = 100$, and $n_2 = 200$

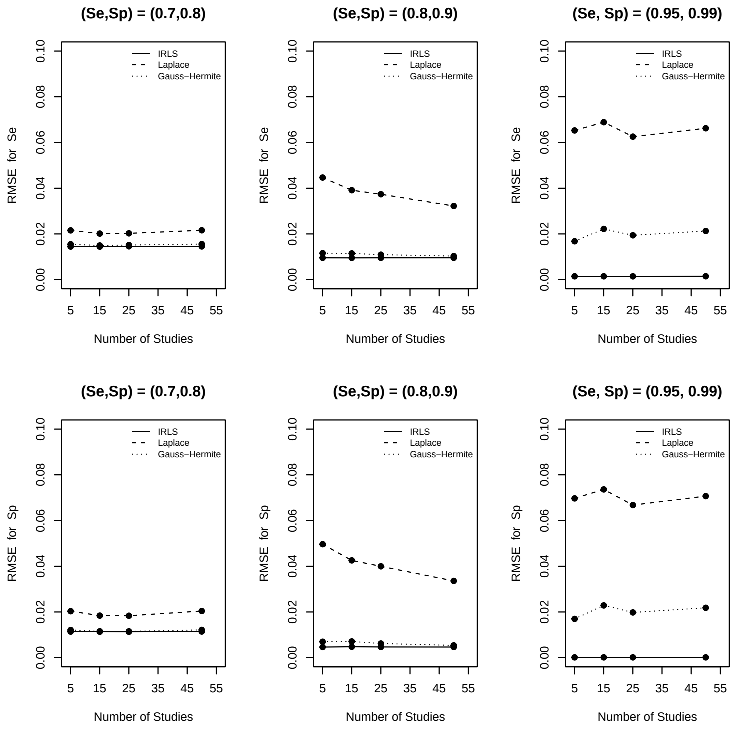

Fig. B11: RMSE for sensitivity (Se) and specificity (Sp) based on the IRWLS (solid line), Laplace approximation (dashed line) and Gauss-Hermite quadrature (dotted line) when $\sigma_1^2 = 1.59$, $\sigma_2^2 = 1.83$, $\sigma_{12} = -0.7$, $n_1 = 50$, and $n_2 = 100$

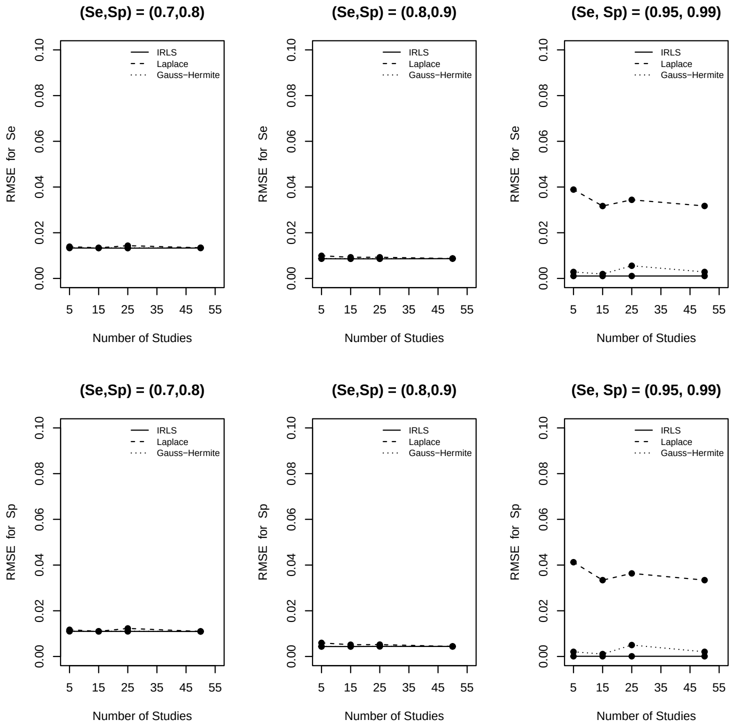

Fig. B12: RMSE for sensitivity (Se) and specificity (Sp) based on the IRWLS (solid line), Laplace approximation (dashed line) and Gauss-Hermite quadrature (dotted line) when $\sigma_1^2 = 1.59$, $\sigma_2^2 = 1.83$, $\sigma_{12} = -0.03$, $n_1 = 300$, and $n_2 = 500$

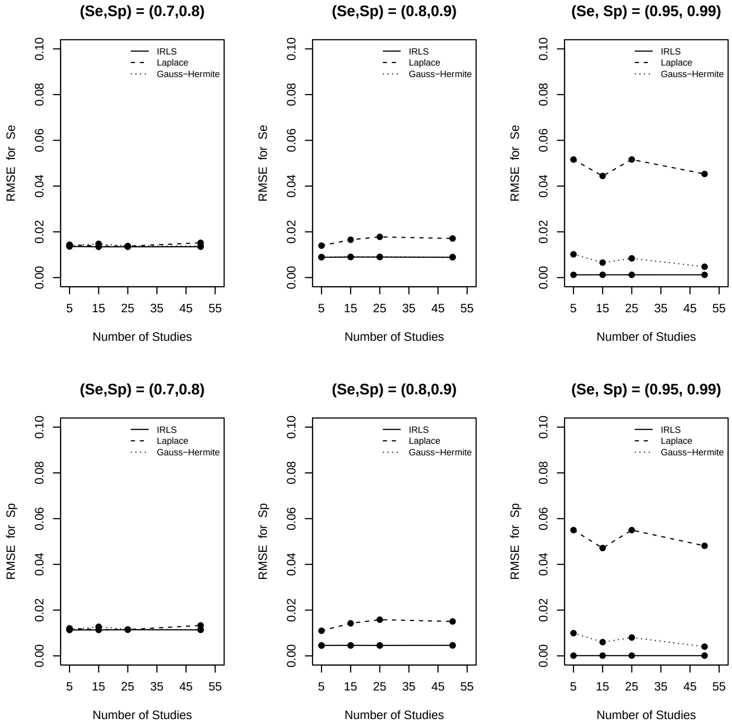

Fig. B13: RMSE for sensitivity (Se) and specificity (Sp) based on the IRWLS (solid line), Laplace approximation (dashed line) and Gauss-Hermite quadrature (dotted line) when $\sigma_1^2 = 1.59$, $\sigma_2^2 = 1.83$, $\sigma_{12} = -0.03$, $n_1 = 100$, and $n_2 = 200$

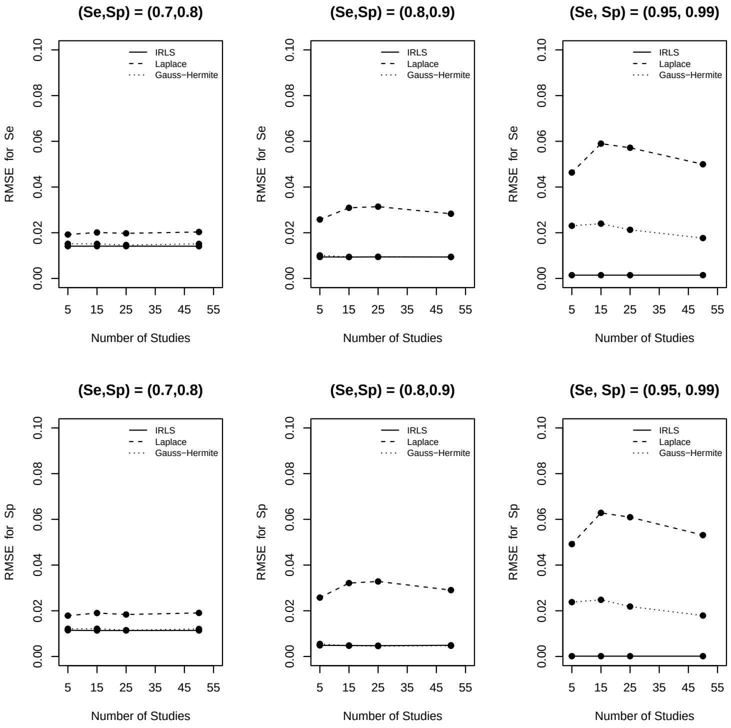

Fig. B14: RMSE for sensitivity (Se) and specificity (Sp) based on the IRWLS (solid line), Laplace approximation (dashed line) and Gauss-Hermite quadrature (dotted line) when $\sigma_1^2 = 1.59$, $\sigma_2^2 = 1.83$, $\sigma_{12} = -0.03$, $n_1 = 50$, and $n_2 = 100$

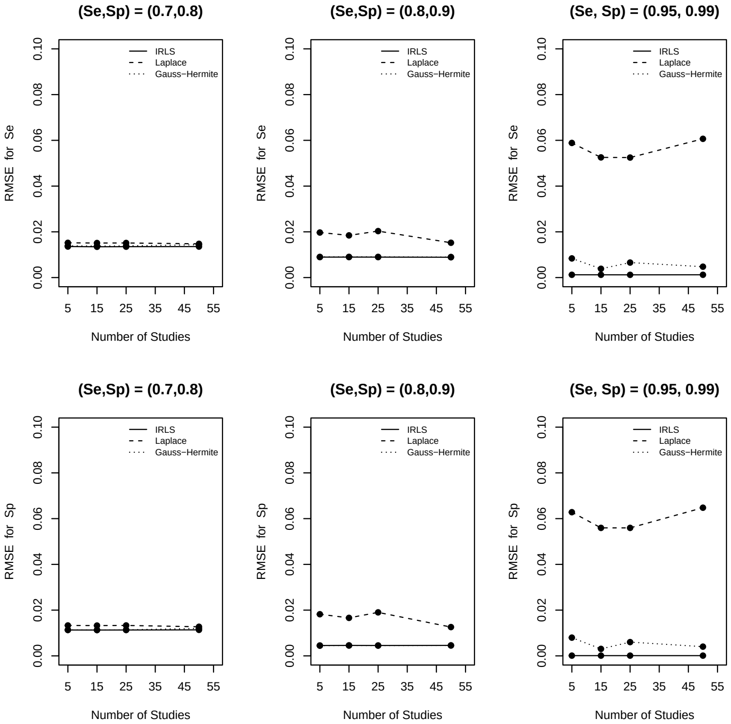

Fig. B15: RMSE for sensitivity (Se) and specificity (Sp) based on the IRWLS (solid line), Laplace approximation (dashed line) and Gauss-Hermite quadrature (dotted line) when $\sigma_1^2 = 1.59$, $\sigma_2^2 = 1.83$, $\sigma_{12} = -0.34$, $n_1 = 100$, and $n_2 = 200$

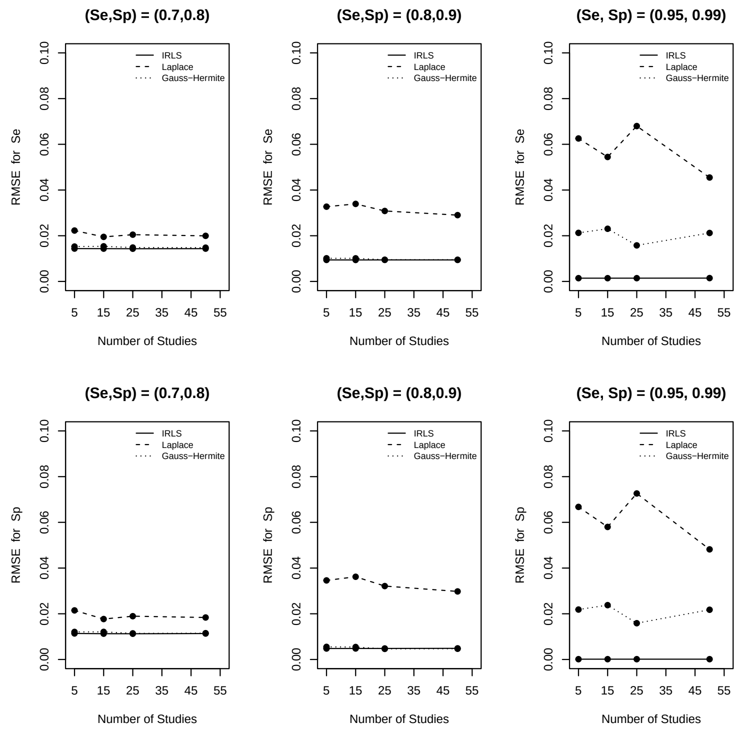

Fig. B16: RMSE for sensitivity (Se) and specificity (Sp) based on the IRWLS (solid line), Laplace approximation (dashed line) and Gauss-Hermite quadrature (dotted line) when $\sigma_1^2 = 1.59$, $\sigma_2^2 = 1.83$, $\sigma_{12} = -0.34$, $n_1 = 50$, and $n_2 = 100$

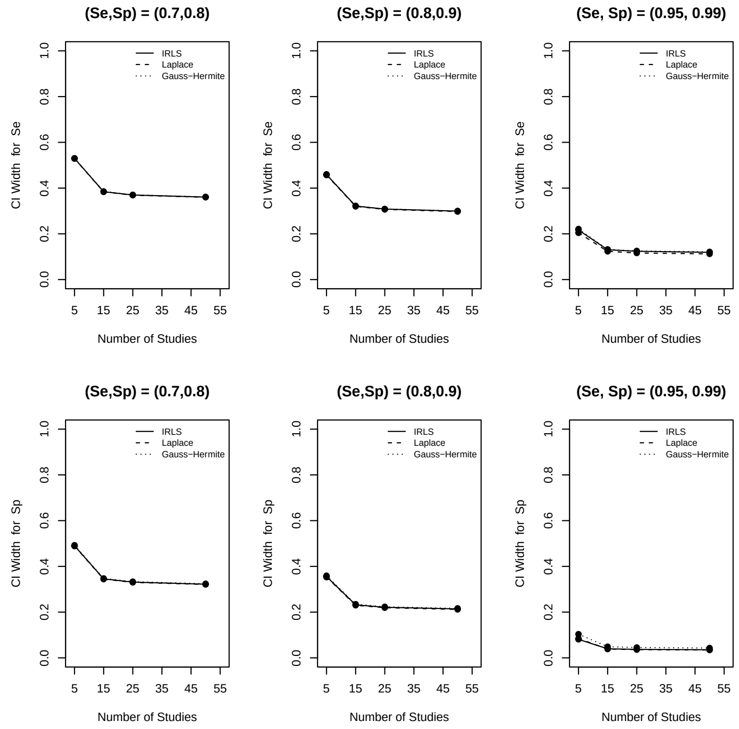

Fig. B17: CI width for sensitivity (Se) and specificity (Sp) based on the IRWLS (solid line), Laplace approximation (dashed line) and Gauss-Hermite quadrature (dotted line) when $\sigma_1^2 = 1.59$, $\sigma_2^2 = 1.83$, $\sigma_{12} = -0.7$, $n_1 = 300$, and $n_2 = 500$

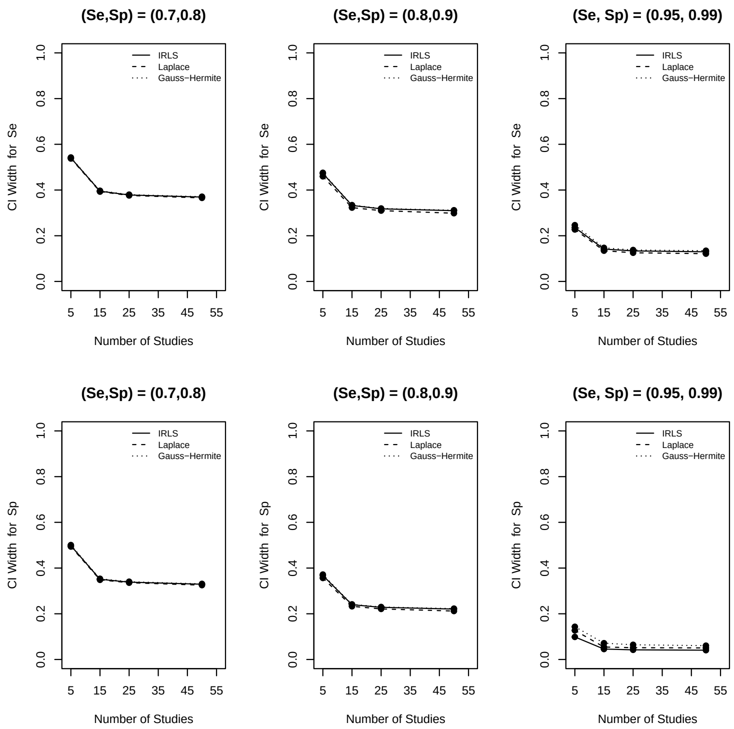

Fig. B18: CI width for sensitivity (Se) and specificity (Sp) based on the IRWLS (solid line), Laplace approximation (dashed line) and Gauss-Hermite quadrature (dotted line) when $\sigma_1^2 = 1.59$, $\sigma_2^2 = 1.83$, $\sigma_{12} = -0.7$, $n_1 = 100$, and $n_2 = 200$

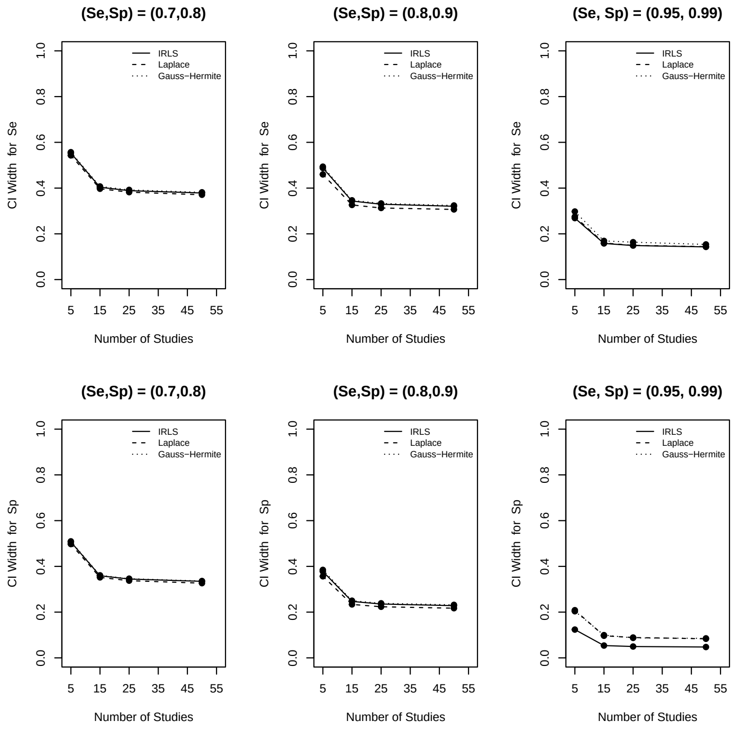

Fig. B19: CI width for sensitivity (Se) and specificity (Sp) based on the IRWLS (solid line), Laplace approximation (dashed line) and Gauss-Hermite quadrature (dotted line) when $\sigma_1^2 = 1.59$, $\sigma_2^2 = 1.83$, $\sigma_{12} = -0.7$, $n_1 = 50$, and $n_2 = 100$

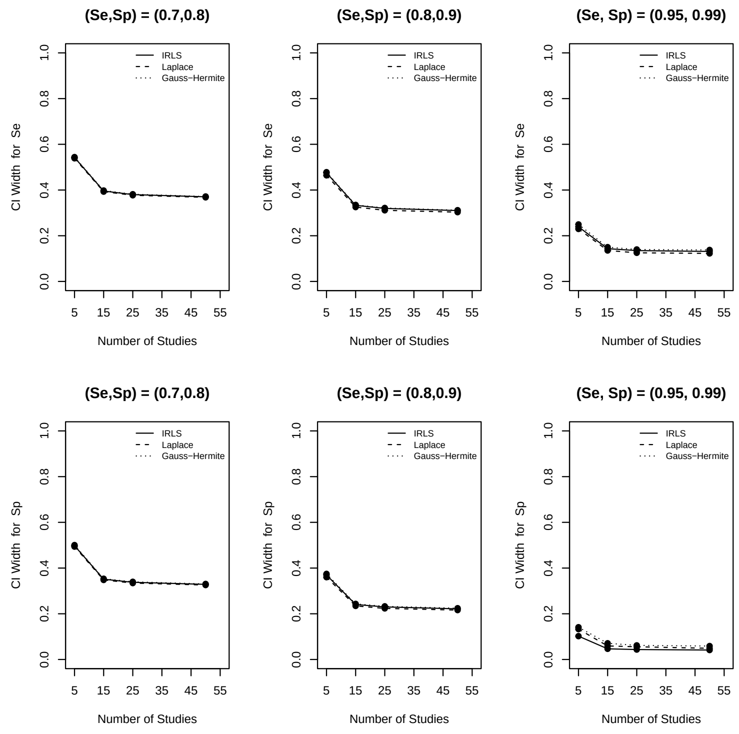

Fig. B20: CI width for sensitivity (Se) and specificity (Sp) based on the IRWLS (solid line), Laplace approximation (dashed line) and Gauss-Hermite quadrature (dotted line) when $\sigma_1^2 = 1.59$, $\sigma_2^2 = 1.83$, $\sigma_{12} = -0.34$, $n_1 = 100$, and $n_2 = 200$

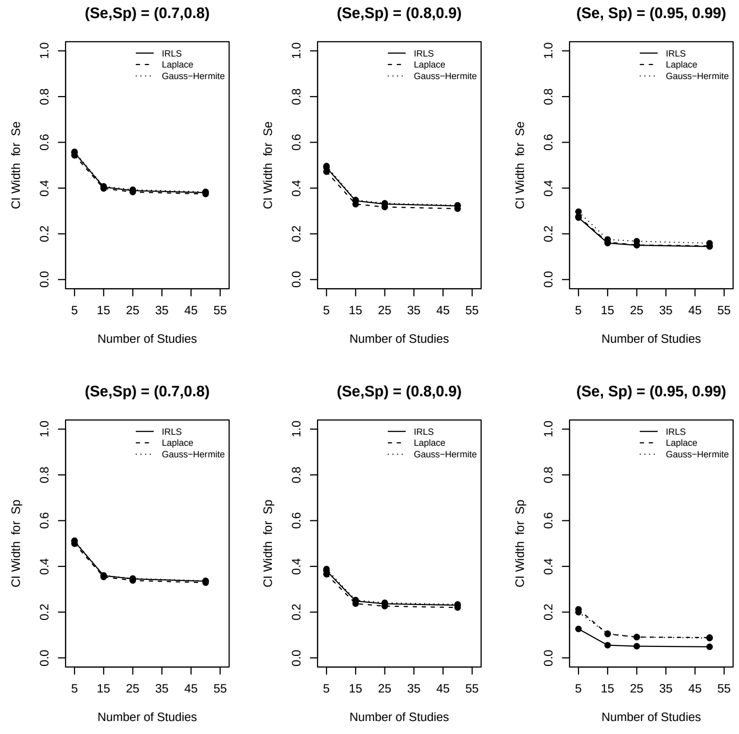

Fig. B21: CI width for sensitivity (Se) and specificity (Sp) based on the IRWLS (solid line), Laplace approximation (dashed line) and Gauss-Hermite quadrature (dotted line) when $\sigma_1^2 = 1.59$, $\sigma_2^2 = 1.83$, $\sigma_{12} = -0.34$, $n_1 = 50$, and $n_2 = 100$

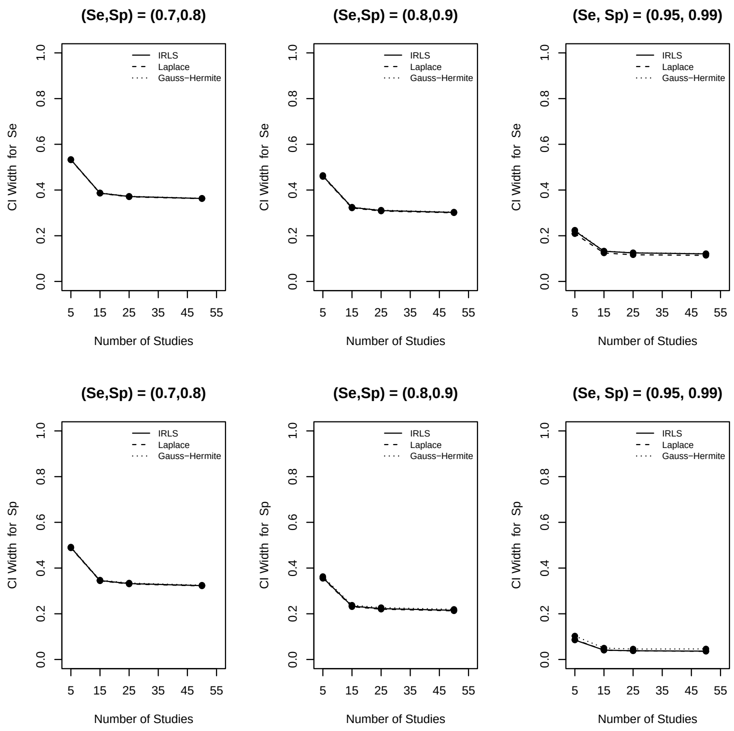

Fig. B22: CI width for sensitivity (Se) and specificity (Sp) based on the IRWLS (solid line), Laplace approximation (dashed line) and Gauss-Hermite quadrature (dotted line) when $\sigma_1^2 = 1.59$, $\sigma_2^2 = 1.83$, $\sigma_{12} = -0.03$, $n_1 = 300$, and $n_2 = 500$

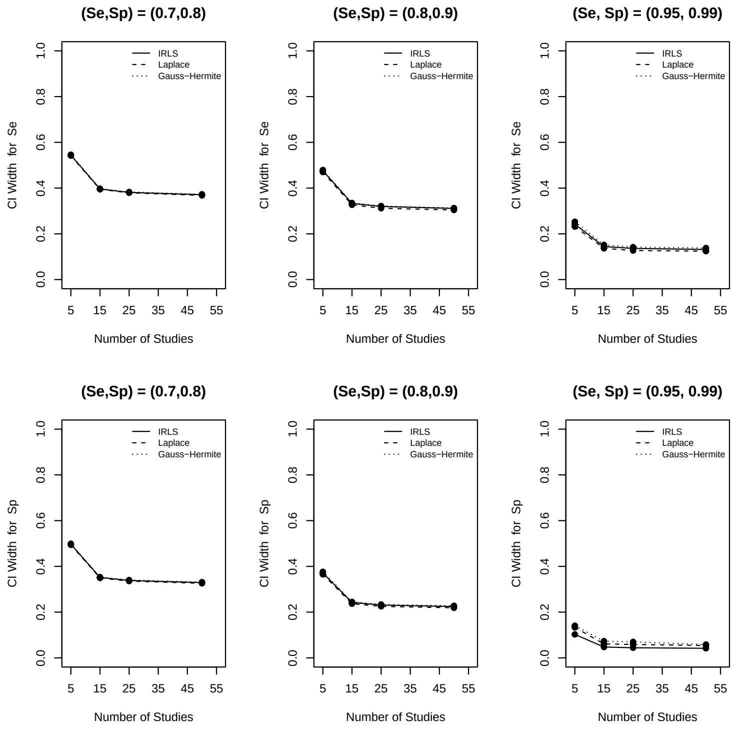

Fig. B23: CI width for sensitivity (Se) and specificity (Sp) based on the IRWLS (solid line), Laplace approximation (dashed line) and Gauss-Hermite quadrature (dotted line) when $\sigma_1^2 = 1.59$, $\sigma_2^2 = 1.83$, $\sigma_{12} = -0.03$, $n_1 = 100$, and $n_2 = 200$

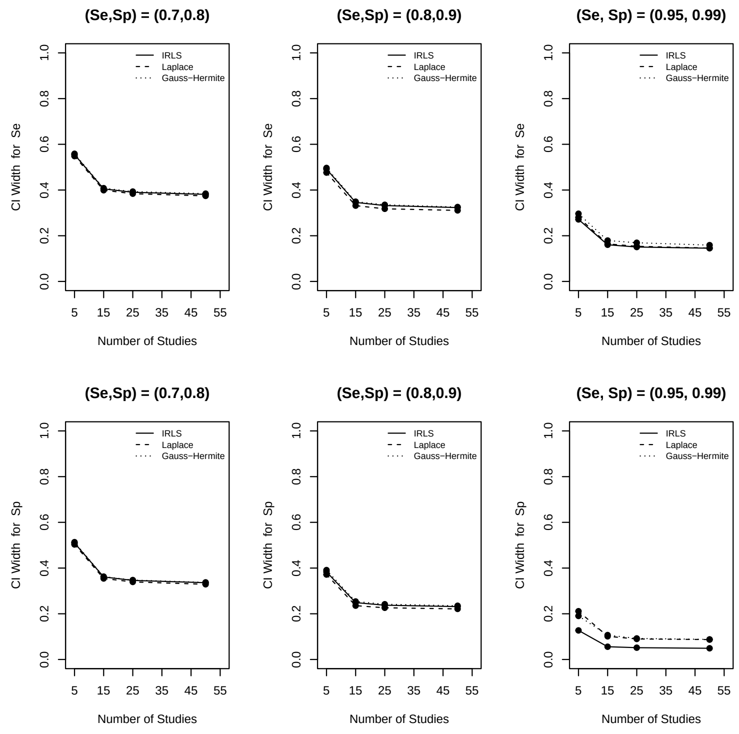

Fig. B24: CI width for sensitivity (Se) and specificity (Sp) based on the IRWLS (solid line), Laplace approximation (dashed line) and Gauss-Hermite quadrature (dotted line) when $\sigma_1^2 = 1.59$, $\sigma_2^2 = 1.83$, $\sigma_{12} = -0.03$, $n_1 = 50$, and $n_2 = 100$

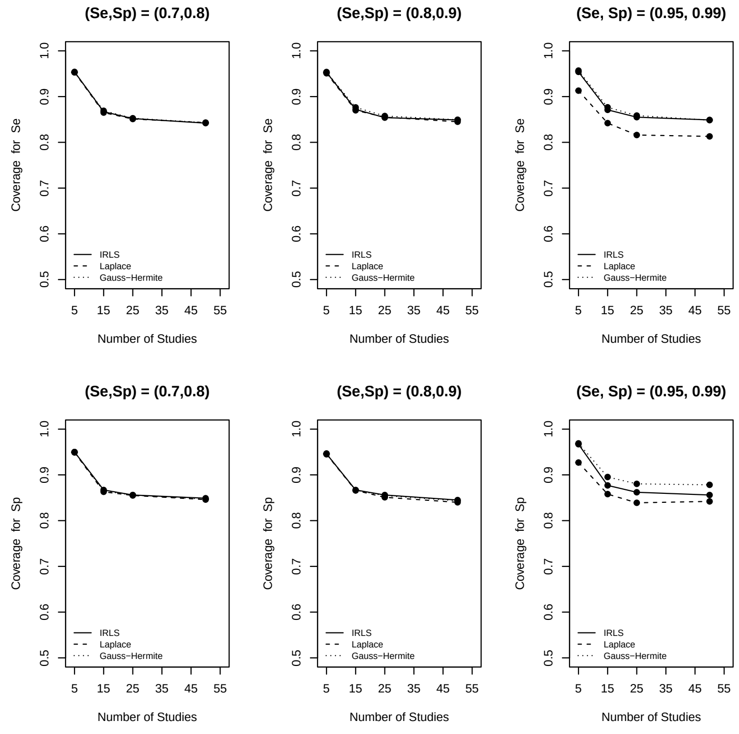

Fig. B25: Coverage for sensitivity (Se) and specificity (Sp) based on the IRWLS (solid line), Laplace approximation (dashed line) and Gauss-Hermite quadrature (dotted line) when $\sigma_1^2 = 1.59$, $\sigma_2^2 = 1.83$, $\sigma_{12} = -0.7$, $n_1 = 300$, and $n_2 = 500$

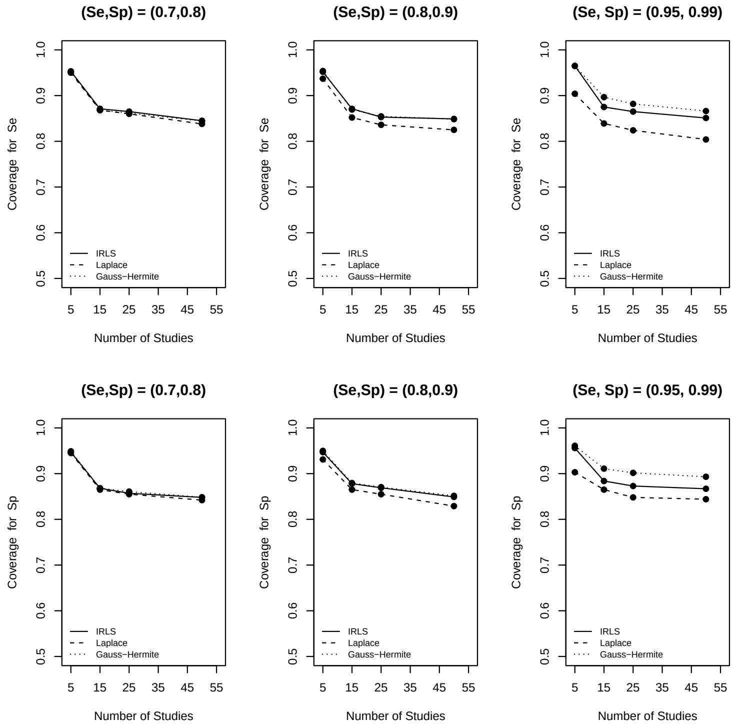

Fig. B26: Coverage for sensitivity (Se) and specificity (Sp) based on the IRWLS (solid line), Laplace approximation (dashed line) and Gauss-Hermite quadrature (dotted line) when $\sigma_1^2 = 1.59$, $\sigma_2^2 = 1.83$, $\sigma_{12} = -0.7$, $n_1 = 100$, and $n_2 = 200$

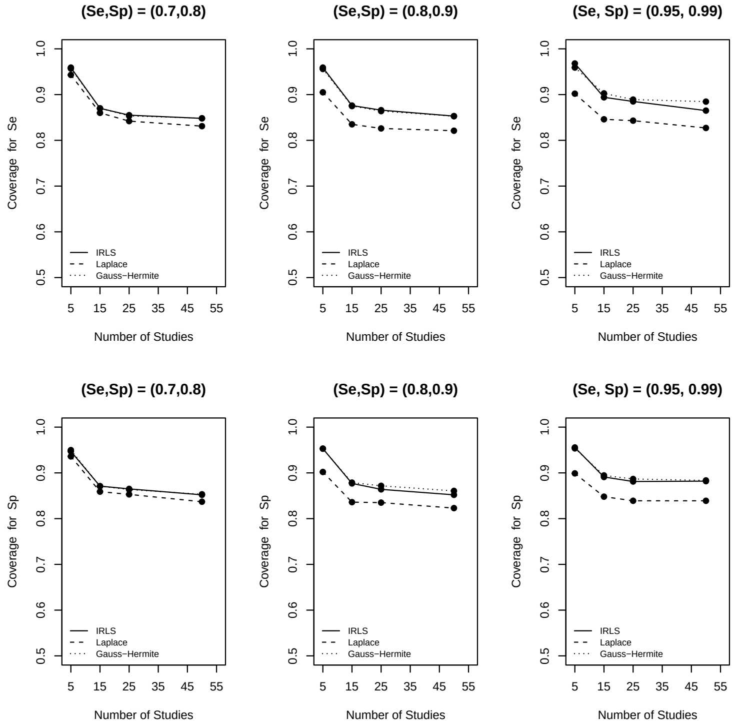

Fig. B27: Coverage for sensitivity (Se) and specificity (Sp) based on the IRWLS (solid line), Laplace approximation (dashed line) and Gauss-Hermite quadrature (dotted line) when $\sigma_1^2 = 1.59$, $\sigma_2^2 = 1.83$, $\sigma_{12} = -0.7$, $n_1 = 50$, and $n_2 = 100$

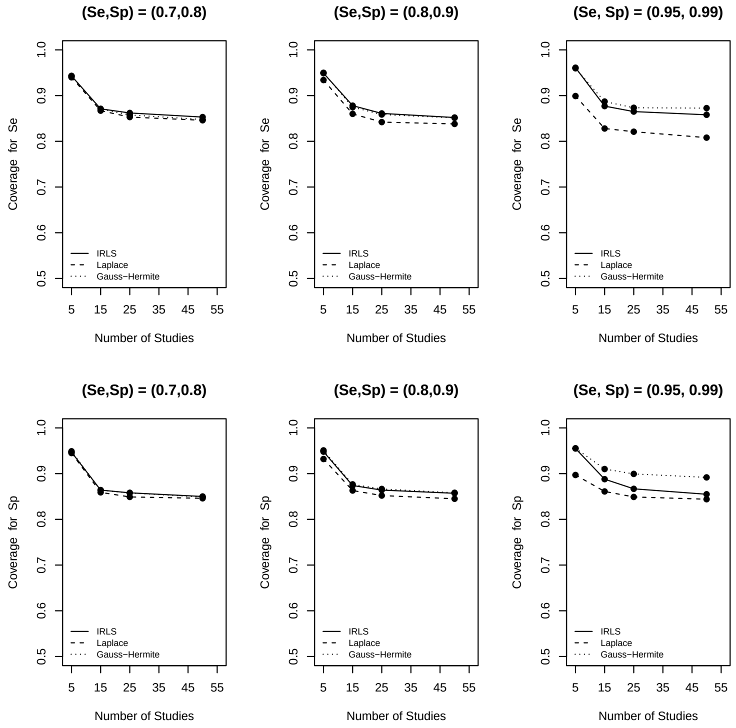

Fig. B28: Coverage for sensitivity (Se) and specificity (Sp) based on the IRWLS (solid line), Laplace approximation (dashed line) and Gauss-Hermite quadrature (dotted line) when $\sigma_1^2 = 1.59$, $\sigma_2^2 = 1.83$, $\sigma_{12} = -0.34$, $n_1 = 100$, and $n_2 = 200$

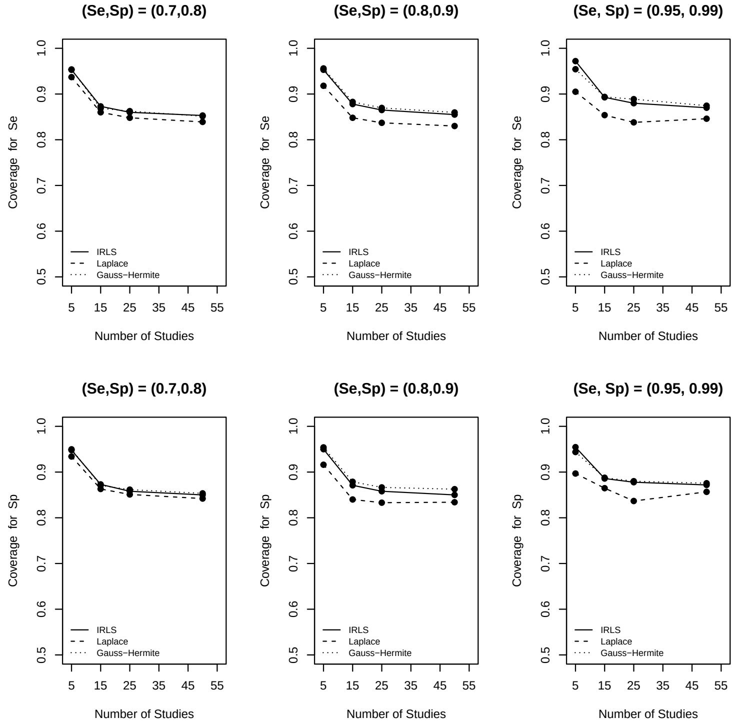

Fig. B29: Coverage for sensitivity (Se) and specificity (Sp) based on the IRWLS (solid line), Laplace approximation (dashed line) and Gauss-Hermite quadrature (dotted line) when $\sigma_1^2 = 1.59$, $\sigma_2^2 = 1.83$, $\sigma_{12} = -0.34$, $n_1 = 50$, and $n_2 = 100$

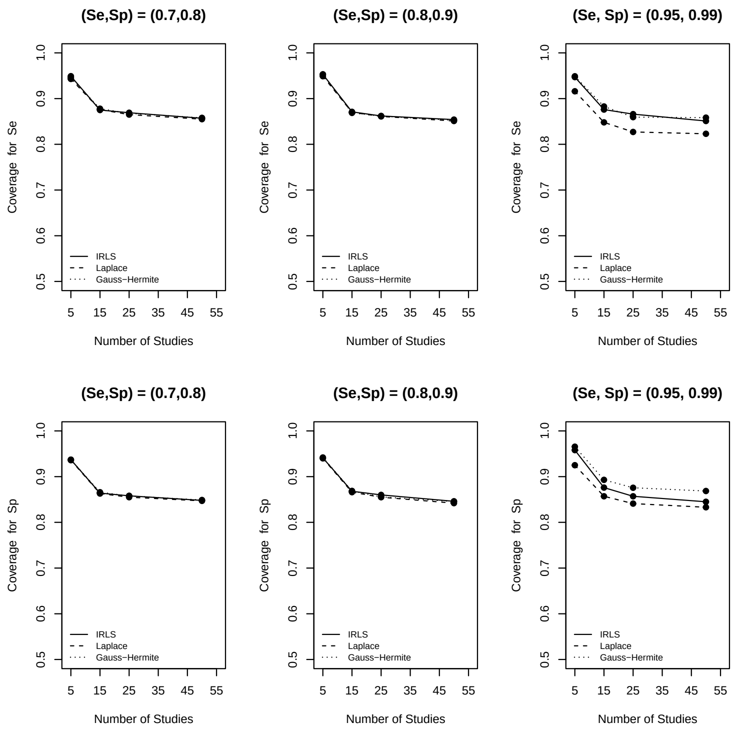

Fig. B30: Coverage for sensitivity (Se) and specificity (Sp) based on the IRWLS (solid line), Laplace approximation (dashed line) and Gauss-Hermite quadrature (dotted line) when $\sigma_1^2 = 1.59$, $\sigma_2^2 = 1.83$, $\sigma_{12} = -0.03$, $n_1 = 300$, and $n_2 = 500$

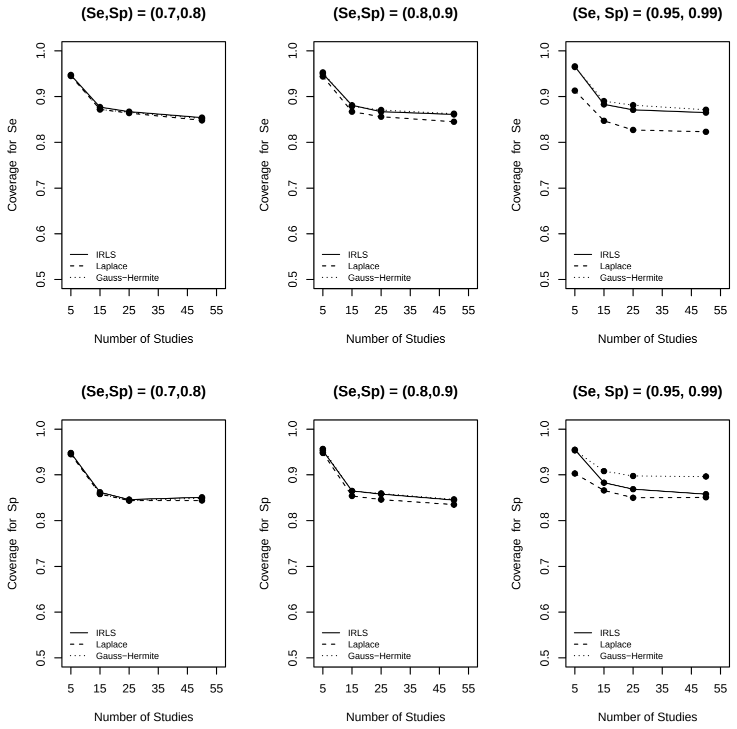

Fig. B31: Coverage for sensitivity (Se) and specificity (Sp) based on the IRWLS (solid line), Laplace approximation (dashed line) and Gauss-Hermite quadrature (dotted line) when $\sigma_1^2 = 1.59$, $\sigma_2^2 = 1.83$, $\sigma_{12} = -0.03$, $n_1 = 100$, and $n_2 = 200$

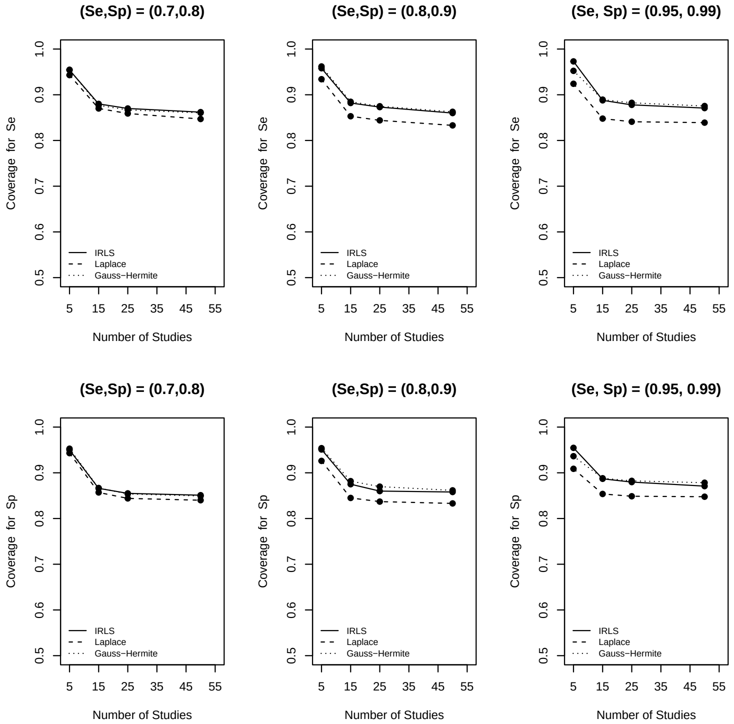

Fig. B32: Coverage for sensitivity (Se) and specificity (Sp) based on the IRWLS (solid line), Laplace approximation (dashed line) and Gauss-Hermite quadrature (dotted line) when $\sigma_1^2 = 1.59$, $\sigma_2^2 = 1.83$, $\sigma_{12} = -0.03$, $n_1 = 50$, and $n_2 = 100$

Appendix C Application of the PQL algorithm to the two motivating meta-analyses

Table C3: Application of the PQL algorithm to the Vonasek et al. (2021) and Jullien et al. (2020) meta-analysis.

Method	Se (95% CI)	Sp (95% CI)	σ_1^2	σ_{12}	σ_2^2	Conv
			Vonasek et al. (2021)			
PQL	0.4866 (0.3633, 0.6115)	0.7201 (0.5753, 0.8302)	0.8431	-0.5990	1.3556	Yes
			Jullien et al. (2020)			
PQL	NA (NA, NA)[a]	0.6951 (0, NA)[b]	5.97×10^{11}	0.0000	41.14×10^{7}	Yes

[a] The back-transformed sensitivity was not available because logit(Se) was unusually large (3.9610×10^{15}). The confidence limits were not available because the standard error of logit(Se) was unusually large (7.1625×10^{13}).

[b] The confidence limits for specificity were not available because the standard error of logit(Sp) was unusually large (2.1316×10^{6}).
